# Upregulation of human PINK1 gene expression by NFκB signalling

**DOI:** 10.1186/s13041-014-0057-y

**Published:** 2014-08-11

**Authors:** Xiaoling Duan, Jade Tong, Qin Xu, Yili Wu, Fang Cai, Tingyu Li, Weihong Song

**Affiliations:** 1 Chongqing City Key Lab of Translational Medical Research in Cognitive Development and Learning and Memory Disorders, and Ministry of Education Key Lab of Child Development and Disorders, Children’s Hospital of Chongqing Medical University, Chongqing 400014, China; 2 Townsend Family Laboratories, Department of Psychiatry, Brain Research Center, The University of British Columbia, 2255 Wesbrook Mall, Vancouver V6T 1Z3, BC, Canada

**Keywords:** PINK1, Mitochondrial function, NFκB, Transcription

## Abstract

Parkinson’s disease (PD) is one of the major neurodegenerative disorders. Mitochondrial malfunction is implicated in PD pathogenesis. Phosphatase and tensin homolog deleted on chromosome 10 (PTEN)-induced putative kinase 1 (PINK1), a serine/threonine kinase, plays an important role in the quality control of mitochondria and more than 70 PINK1 mutations have been identified to cause early-onset PD. However, the regulation of PINK1 gene expression remains elusive. In the present study, we identified the transcription start site (TSS) of the human PINK1 gene using switching mechanism at 5’end of RNA transcription (SMART RACE) assay. The TSS is located at 91 bp upstream of the translation start site ATG. The region with 104 bp was identified as the minimal promoter region by deletion analysis followed by dual luciferase assay. Four functional cis-acting nuclear factor kappa-light-chain-enhancer of activated B cells (NFκB)-binding sites within the PINK1 promoter were identified. NFκB overexpression led to the up-regulation of PINK1 expression in both HEK293 cells and SH-SY5Y cells. Consistently, lipopolysaccharide (LPS), a strong activator of NFκB, significantly increased PINK1 expression in SH-SY5Y cells. Taken together, our results clearly suggested that PINK1 expression is tightly regulated at its transcription level and NFκB is a positive regulator for PINK1 expression.

## Background

Parkinson’s disease (PD) is the second most common neurodegenerative disease [1]. PD patients commonly suffer from muscular dysfunction which resulted in body rigidity, tremors, bradykinesia, posture instability, and Parkinsonian gait. Secondary symptoms such as anxiety, depression, memory loss, and dementia may appear over the course of disease progression [2],[3]. The pathological hallmarks of PD include neurodegeneration of dopaminergic neurons specifically in the substantia nigra [3], and intracellular aggregation of misfolded proteins forming lewy bodies [4]. Although clinical and experimental studies suggest the involvement of protein misfolding, oxidative stress and mitochondrial dysfunction, the fundamental cause of the disease, its underlying mechanism remains elusive.

Phosphatase and tensin homolog deleted on chromosome 10 (PTEN)-induced putative kinase 1 (PINK1), initially was identified as a downstream molecule of PTEN in cancer cells [5]. PINK1 is a type I transmembrane protein of 581 amino acids with a putative serine/threonine kinase domain and an N-terminal mitochondrial targeting signal. Upon synthesis, the full-length PINK1 (FL-PINK1) is imported into mitochondria with the C-terminus facing the cytosol and undergoes proteolysis to produce a predominant product of ~55 kDa (∆1-PINK1) and a minor one of ~45 kDa (∆2-PINK1) [6],[7]. The proteolytic process is mediated by matrix processing peptidase and presenilin-associated rhomboid-like protein (PARL) [8]. ∆1-PINK1 was reported to be released to the cytosol and interacts with Parkin, a PD-associated E3 ubiquitin ligase [9]. The binding of ∆1-PINK1 with Parkin impairs the recruitment of Parkin to mitochondria and leads to the degradation of Parkin and PINK1 by proteasome pathway in healthy mitochondria [9]. Parkin, PINK1 and DJ-1 formed a complex to promote ubiquitination and degradation of Parkin substrates, including Parkin itself and Synphilin-1 [10]. However, in dysfunctional mitochondria with reduced mitochondrial membrane potential and mitochondrial oxidative stress, full-length PINK1 is aggregated on the mitochondrial membrane and recruits Parkin to mitochondria through the phosphorylation of Parkin, resulting in mitochondrial autophagy. PINK1 deficiency reduces mitochondrial membrane potential and compromises complex I activity of the mitochondria respiratory chain [11]. Studies suggest that PINK1 together with Parkin is critical for the quality control of mitochondria, including mitochondrial integrity, turnover, and functions. More than 70 mutations in the PINK1 gene were identified in familial PD in an autosomal recessive manner [12],[13]. Most of these mutations fail to phosphorylate and recruit Parkin to mitochondria, leading to the accumulation of dysfunctional mitochondria and eventually neuronal death. This strongly suggests that PINK1 plays a critical role in PD pathogenesis and dysregulation of PINK1 may contribute to the development of PD.

Nuclear factor kappa-light-chain-enhancer of activated B cells (NFκB) is a family of diametric transcription factors, regulating numerous genes involving in cell survival, inflammation and immunity. The NFκB family consists of five members, p50, p52, p65 (RelA), RelB and C-Rel. All of them share an N-terminal Rel homology domain (RHD) responsible for DNA binding and homo- or hetero-dimerization. Only three members including p65, RelB and C-Rel contain transcription activation domain (TAD) necessary for the activation of target genes. NFκB exists in forms of homo- or hetero-dimer and the most abundant form is the p65/p50 heterodimer. NFκB dimers bind with inhibitor of κB (IκB) proteins in cytoplasm, preventing its nuclear translocation and DNA binding. Once IκB protein degradation is induced, NFκB dimers are released from IκB-NFκB complex and subsequently translocated from cytoplasm to nucleus [14], thus transcription regulation of its target genes is initialized [15]. NFκB can be activated by stimuli such as oxidative stress, cytokines, free radicals and biological antigens. Oxidative stress or free radicals has been shown to be involved in PD. An increase of NFκB translocation to the nucleus and activation have been observed in dopaminergic neurons from both PD animal models and patient samples as well as other neurodegenerative disorders [16]-[21]. It has been shown that NFκB plays an important role in regulating PD related genes, such as UCHL1 [22], USP24 [23] and RNF11 [24].

Although the function of PINK1 is well studied in terms of mitochondrial quality control, the regulation of PINK1 gene expression is barely explored. In the present study, we aimed to understand the transcriptional regulation of the PINK1 gene. For the first time, we identified that the transcription start site (TSS) of PINK1 is located 91 bp upstream of the translation start site (ATG). The region of 104 bp (−78 to +26) contains the minimal promoter with functional transcriptional activity for the PINK1 gene. Furthermore, we showed that the PINK1 gene promoter contains 4 functional cis-acting NFκB-binding sites, which promote PINK1 expression. Taken together, our results demonstrated that PINK1 expression is tightly regulated at its transcriptional level and that NFκB is a positive regulator for PINK1 expression.

## Results and discussion

### Cloning the human *PINK1* gene promoter and mapping its transcription initiation site

**Figure 1:**
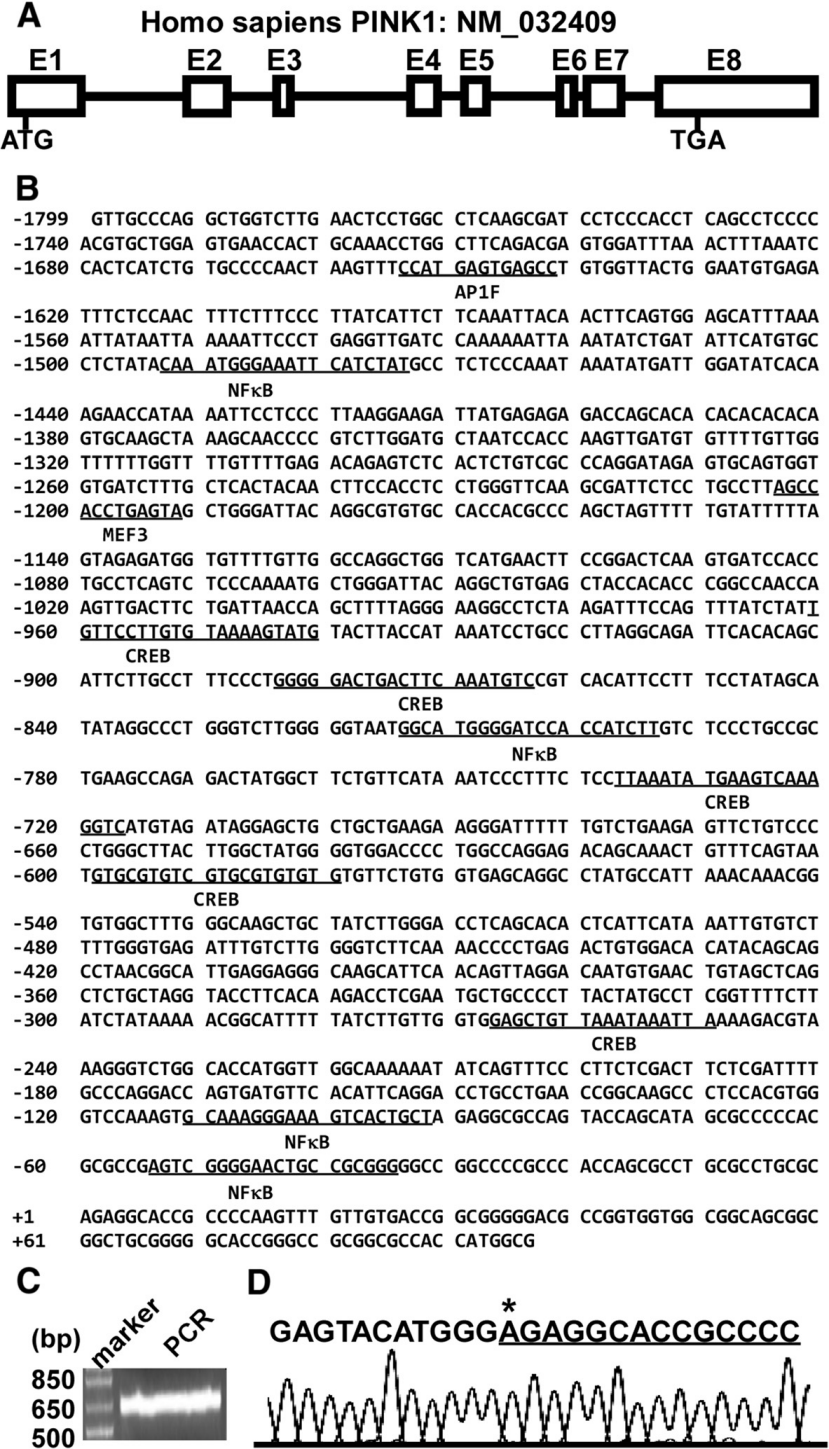
**Sequence features of the human PINK1 gene promoter. (A)** The genomic organization of human PINK1 gene on chromosome 1. E represents exon. ATG is the translation start codon and TGA is the stop codon. **(B)** The nucleotide sequence of the human PINK1 gene from −1799 to +97 bp. The adenine +1 represents the transcription start site. The putative transcription factor binding sites are underlined in bold face. **(C)** SMARTer-RACE was performed to map the PINK1 transcription start site. The PCR product was run on a 1.5% agarose gel. **(D)** The PCR product was cloned into pcDNA4 vector and sequenced to identify the transcription start site. The first base after the adapter is the TSS from which is underlined.

The human PINK1 gene spans a region of 18,056 bp on chromosome 1p36. The human PINK1 gene transcript (NCBI Reference Sequence: NM_032409) is 2680-bp long and composed of 8 exons (Figure 1A). Human genomic DNA samples were extracted from SH-SY5Y cells and an 1825 bp 5’ flanking region of the PINK1 gene was amplified and cloned. The DNA fragment was sequenced (Figure 1B). To identify the transcription start site of human PINK1 gene, SMART-RACE was performed. The 5’ends of PINK1 cDNA with approximate 650 bp in length were amplified (Figure 1C). The sequencing results indicated that the transcription start site (TSS), an adenine labeled as +1, is located 91 bp upstream of the translation start site (Figure 1D). Further sequence analysis and a computer-based transcription factor binding site search using Genomatix and TFSearch revealed that the human PINK1 gene has a complex transcriptional unit. The human PINK1 gene promoter lacks typical TATA and CAAT boxes, but contains several putative regulatory elements, such as AP1, MEF, and cAMP-responsive elements (Figure 1B).

### Functional characterization of the PINK1 promoter

**Figure 2:**
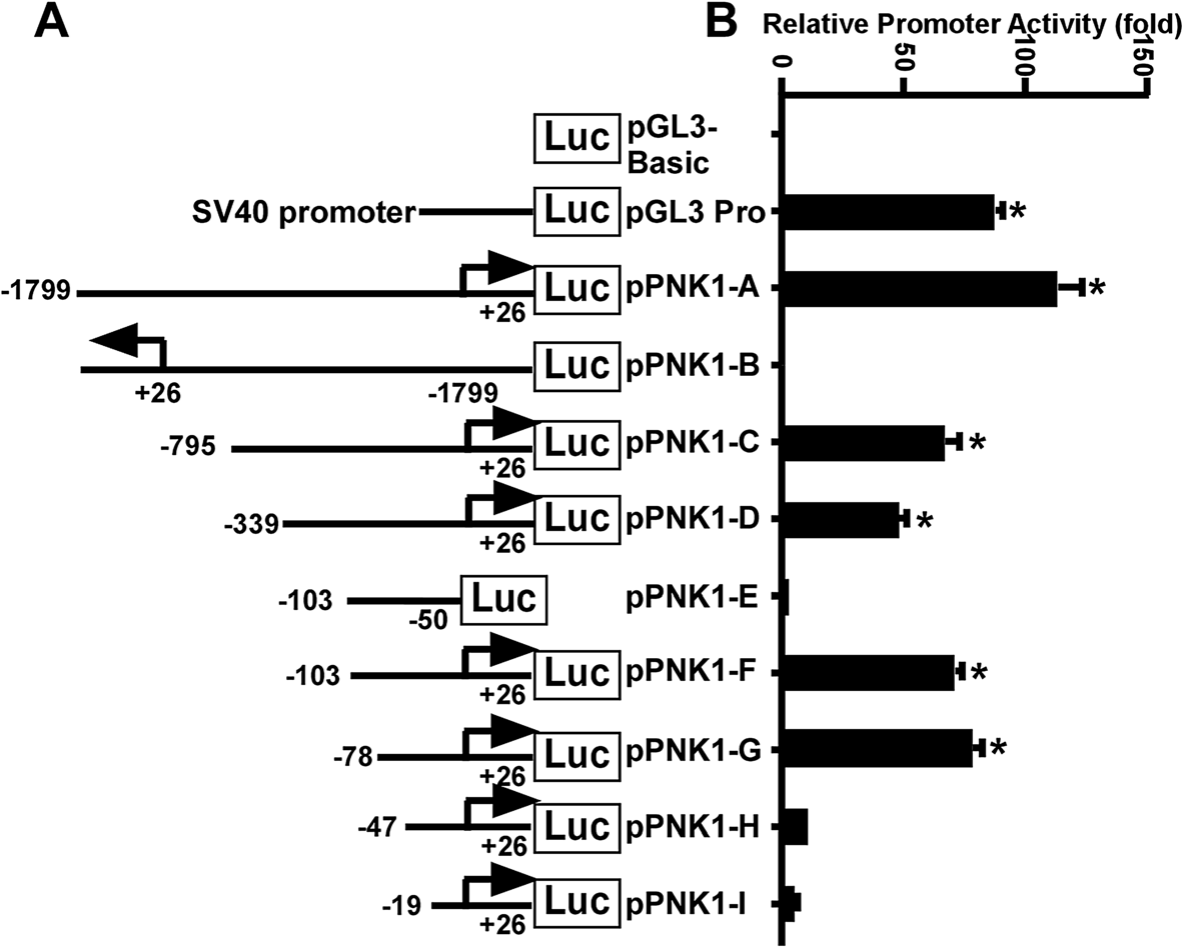
**Deletion analysis of the human PINK1 gene promoter. (A)** Schematic diagram of the PINK1 promoter constructs consisting of the 5’ flanking region with serial deletions cloned into the pGL3-basic vector. Arrow shows the direction of transcription. The numbers represents the end points of each construct. **(B)** The deletion plasmids were cotransfected with pCMV-Luc into HEK293 cells. 24 h after the transfection, the luciferase activity was measured and expressed in relative luciferase units (RLU). The pCMV-Luc was used to normalize for transfection efficiency. The values represent means ± SEM. n = 3, *p < 0.0001, by one-way ANOVA followed by post hoc Tukey’s multiple comparisons test.

To determine the functional promoter region of PINK1 gene, we sub-cloned the 1,825 bp of 5’ flanking region of the PINK1 gene into the promoterless plasmid vector pGL3-basic. The pGL3-basic vector lacks eukaryotic promoter and enhancer sequences upstream of a reporter luciferase gene. The luciferase activity in cells transfected with this plasmid depends on the presence and proper orientation of a functional promoter upstream of the luciferase gene. The pPINK1-A plasmid was constructed to contain the 1,825 bp fragment from −1799 to +26 bp of PINK1 gene upstream of the luciferase reporter gene. Plasmid DNA was transfected into HEK293 cells, and luciferase activity was measured by a luminometer to reflect promoter activity. Compared with cells transfected with an empty vector pGL3-basic, pPINK1-A-transfected cells showed a robust luciferase activity (113.47 ± 10.63 RLU) (Figure 2B). This result indicated that the 1,825 bp fragment contains the functional promoter region of the human PINK1 gene. To further identify the minimal promoter region required for PINK1 gene expression, a series of deletion mutants within the 1,825 bp fragment of pPINK1-A were generated as indicated in Figure 2A. The luciferase activity assays of these deletion mutants were performed. The results indicated that placing the 1,825 bp fragment in a reverse orientation (pPINK1-B) completely abolished luciferase activities observed in pPINK1-A transfected cells (Figure 2B). Deletion of −1799 to -103 bp in pPINK1-F reduced the luciferase activities of pPINK1-A moderately (71.16 ± 3.02 RLU). Deletion of the identified transcription initiation site (pPINK1-E) from pPINK1-F occluded the remaining luciferase activities (Figure 2B). Further deletion of the promoter region to −78 to +26 bp maintained similar luciferase activity in pPINK1-G (78.44 ± 4.74 RLU) comparing to pPINK1-F (*p* > 0.05). However, additional deletion of 31 bp from pPINK1-G to pPINK1-H (−47 to +26) significantly reduced the luciferase activity to 11.46 ± 2.50 RLU (*p* < 0.05). These data suggest that the region from −78 to +26, containing the transcription start site, possesses the minimal promoter activity.

### The human PINK1 gene promoter contains NFκB binding sites

**Figure 3:**
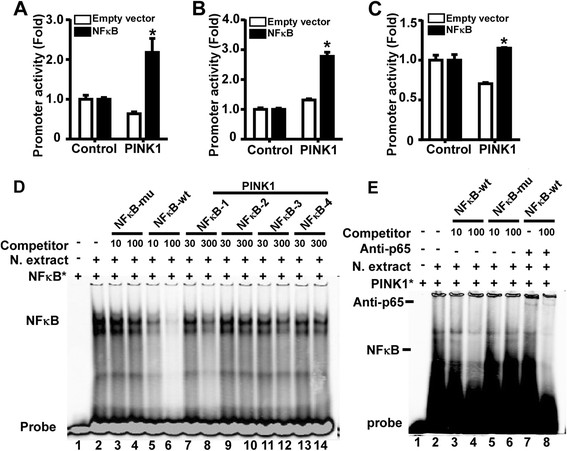
**NFκB binds to human PINK1 gene promoter.** The effects of NFκB on the human PINK1 promoter in HEK293 cells **(A)**, SH-SY5Y cells **(B)** and N2A cells **(C)** were analyzed by luciferase reporter assays. The PINK1 promoter reporter plasmid (pPINK1-A) or pGL3-promoter was co-transfected into cells with NFκB expression plasmid or the empty vector pMTF. Values indicate means ± SEM. n = 3, *p < 0.01 by Student’s *t*-test.**(D)** EMSA with NFκB p65 consensus probe. Lane 1 is labeled probe alone without protein extract. Incubation of the probe with NFκB p65 enriched nuclear extracts forms a shifted DNA-protein complex band (lane 2). Competition assays were performed by further adding different competition oligonucleotides including NFκB-mu, NFκB-wt, and PINK1-NFκB. **(E)** EMSA with PINK1 NFκB-1 probe. Lane1 is labelled IR700-PINK1-NFκB-1 probe only. Addition of NFκB p65 enriched nuclear extracts forms a shifted DNA-protein complex band (lane 2). Lane3 to 6, the competitor NFκB-wt and NFκB-mu were added at 10- or 100-fold. Lane 7 and 8, addition of anti-p65 antibody forms a super shifted DNA-protein-antibody band (lane7) and NFκB-wt was added for competition (lane 8).

Computer-based transcription factor binding site analysis revealed four putative NFκB elements in the 1.8 kb promoter region of the human PINK1 gene (Figure 1B). To determine whether NFκB signaling regulates PINK1 gene transcription by interacting with these putative NFκB *cis*-acting elements, the effect of NFκB overexpression on the promoter activity of the 1.8 kb region were examined. Plasmid pPINK1-A, containing 4 NFκB binding sites, was transfected into HEK293 cells, SH-SY5Y cells or N2A cells with either the NFκB expression plasmid or the empty vector pMTF. The results showed that NFκB overexpression significantly increased PINK1 promoter activity to 3.43 ± 0.55 folds (Figure 3A), 2.11 ± 0.10 folds (Figure 3B) and 1.63 ± 0.01 folds (Figure 3C) in HEK293, SH-SY5Y cells and N2A cells, respectively (*p* < 0.01). These results suggest that NFκB activates PINK1 gene transcription and the PINK1 promoter fragment contains functional NFκB sites.

The four putative NFκB elements in the 1.8-kb PINK1 promoter region are located at −1493 to -1474 bp, −814 to -794 bp, −111 to -92 bp, and −54 to -35 bp, respectively. The sequences of these regions are highly homologous to the NFκB consensus sequence 5’-GGGRNNYYCC in which R stands for purine, Y stands for pyrimidine, and N stands for deoxynucleotides. To determine which putative binding site actually binds with NFκB, EMSA was performed using four pairs of synthesized oligonucleotides containing the binding sequence. The sequences of these four oligonucleotides are: PINK1-NFκB-1, 5’- caaatgggaaattcatctat, PINK1-NFκB-2, 5’- ggcatggggatccaccatctt, PINK1-NFκB-3, 5’- gcaaagggaaagtcactgct, and PINK1-NFκB-4, 5’- agtcggggaactgccgcggg, respectively. A NFκB binding probe (NFκB-Wt-oligo) served as a positive binding probe and a mutant NFκB binding probe (NFκB-Mu-oligo) which loses the NFκB binding ability served as a negative binding probe [25]. NFκB-Wt-oligo was labelled with IRDye-700 and the labelled probe was named NFκBwt-IR700. After 30-min incubation with NFκB enriched nuclear extracts of HEK293 cells, the NFκBwt-IR700 bound to NFκB protein and formed a shifted DNA-protein complex band (Figure 3D, lane2). Adding 10-fold of the unlabelled NFκB-Wt-oligo sharply reduced the intensity of the shifted band, indicating that the unlabelled NFκB-Wt-oligo competed with and reduced the binding of NFκB-wt-IR700 to NFκB protein (Figure 3D, lane 5). Increasing the amount of unlabelled NFκB-Wt-oligo to 100-fold almost completely abolished the shifted band (Figure 3D, lane 6). Addition of the NFκB-Mu-oligo, which fails to bind with NFκB, did not affect the interaction between NFκBwt-IR700 and protein (Figure 3D, lane 3–4). The PINK-NFκB-1 to −4 oligos were tested for their ability to compete against NFκBwt-IR700 binding with NFκB protein. The results indicated that addition of 30-fold excess of the four PINK1-NFκB oligos individually barely reduced the intensity of the shifted band (Figure 3D, lane 7, 9, 11, 13). However, increasing the amount to 300-fold significantly reduced the intensity of the shifted band for PINK1-NFκB-1 and PINK1-NFκB-3, with PINK1-NFκB-1 being a stronger competitor than PINK1-NFκB-3 in binding with NFκB protein (Figure 3D, lane 8 and 12). PINK1-NFκB-2 and PINK1-NFκB-4 showed limited ability in competing with NFκBwt-IR700 to bind with NFκB (Figure 3D, lane 10 and 14).

To further confirm the binding between PINK1-NFκB-1 in the human PINK1 promoter region and NFκB, we performed EMSA with IRDye-700 labelled PINK1-NFκB-1. The probe showed a shifted band (Figure 3E lane 2) after incubation with nuclear extracts from HEK293 cells over-expressing NFκB. The intensity of the shifted band was remarkably reduced when a 10-fold excess of NFκB-Wt-oligo was added to the incubation and abolished after the concentration of the competitor increased to 100-fold (Figure 3E, lane3-4). However, no competition was found when either 10-fold or 100-fold NFκB-Mu-oligo was added (Figure 3E, lane 5 and 6). A super-shift assay was further performed with the anti-p65 antibody. The super-shifted band containing labelled PINK1-NFκB-1, NFκB, and anti-p65 antibody was observed at the top of the gel (Figure 3E, lane 7), and the band was competed down by NFκB-Wt-oligo (Figure 3E lane 8). Taken together, the EMSA results demonstrate that NFκB binds to the human PINK1 gene promoter and the −1493 to -1474 bp of PINK1 promoter region is the main binding site.

### NFκB increases the human PINK1 gene expression

**Figure 4:**
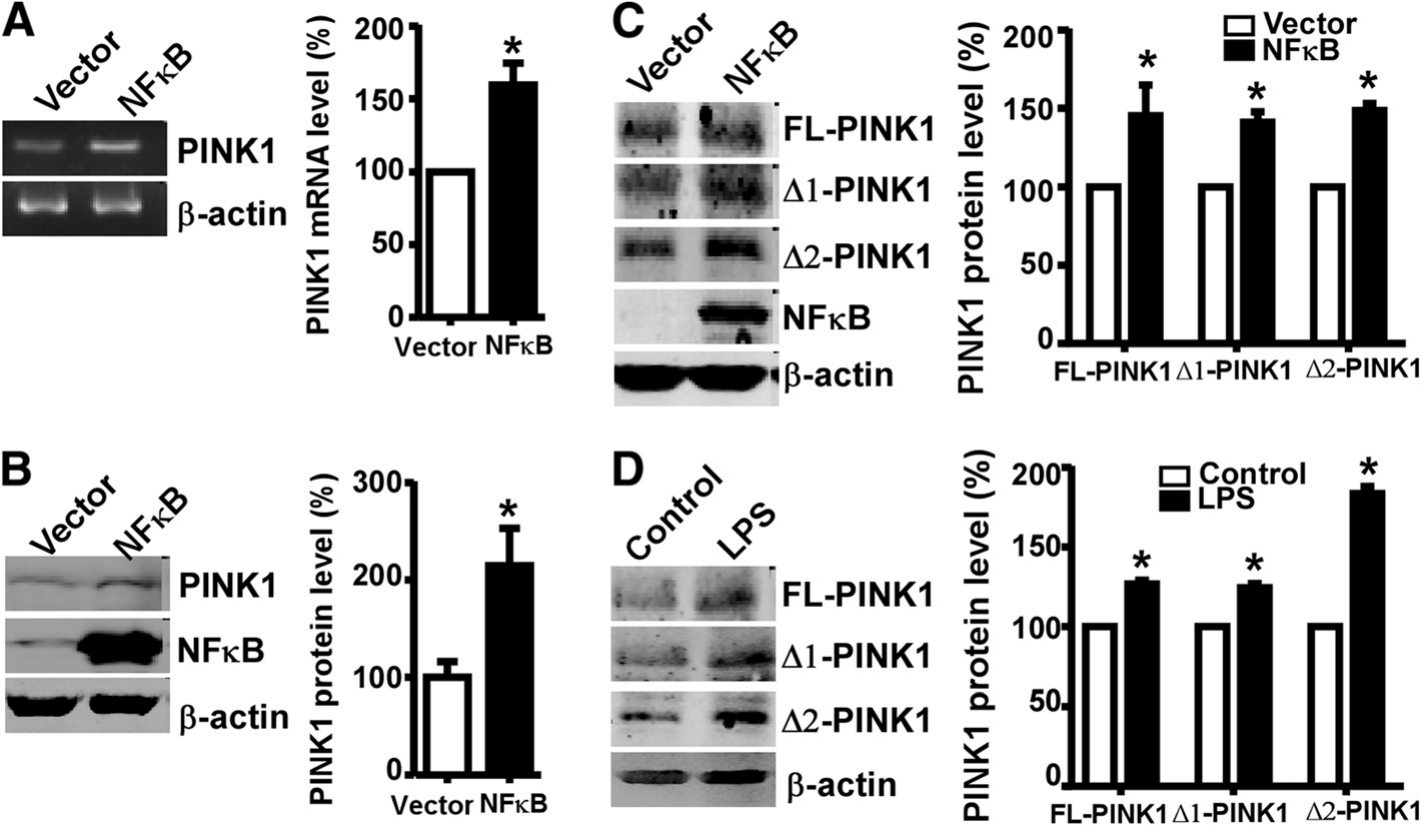
**NFκB upregulates human PINK1 gene expression. (A)** NFκB increases the endogenous human PINK1 mRNA level. HEK293 cells were transfected with either the NFκB expression vector or empty vector pMTF. RT-PCRs were performed using either primers specific to the human PINK1 coding sequence or the human β-actin coding sequence. **(B)** NFκB increases the endogenous human PINK1 protein levels. HEK293 cells transfected with NFκB were harvested 48 h after transfection for protein detection. Cell lysates were run on 10% glycine gel and images were collected by Licor. A significant increase of endogenous PINK1 was observed. **(C)** The endogenous human PINK1 protein level was dramatically increased by NFκB in SH-SY5Y cells. SH-SY5Y cells were transfected with NFκB p65 plasmids or the control vector pMTF and then harvested for protein detections. The human PINK1 protein and its proteolysis products ∆1-PINK1 (55kD) and ∆2-PINK1 (45kD) were all increased. β-actin acted as the internal control. **(D)** LPS treatment facilitated PINK1 expression in SH-SY5Y cells. Cells were harvested after being treated for 16 h and then subjected to Western blot. The human β-actin level was served as a control. Quantification was performed by Image J software. Values indicate means ± SEM. n = 3, *p <0.01 by Student’s *t*-test.

Next we examined the effects of NFκB on PINK1 gene expression by RT-PCR and Western blot analysis. The semi-quantitative RT-PCR was performed to test whether endogenous PINK1 mRNA level was affected by NFκB. Using the human β-actin as an internal control, NFκB overexpression significantly increased endogenous human PINK1 transcription by 59.51% ± 15.13% (*p* < 0.01) (Figure 4A). To examine whether NFκB modulates PINK1 expression at the protein level, endogenous PINK1 protein was detected by Western blot analysis. The full-length human PINK1 protein (FL-PINK1, 63 kDa) and its proteolytic products, ∆1-PINK1 (55 kDa) and ∆2-PINK1 (45 kDa), were detected and analyzed. The endogenous PINK1 expression increased by 114.53% ± 38% (*p* < 0.01) (Figure 4B) in the NFκB overexpressing HEK293 cells when compared with the vector control. Meanwhile, the same experiment was performed in SH-SY5Y cells and three PINK1 species were observed. Consistently, the endogenous PINK1 bands were also up-regulated in NFκB overexpressing SH-SY5Y cells by 45.26% ± 19.58%, 41.10 ± 6.70% and 48.54 ± 4.50% (*p* < 0.01), respectively (Figure 4C). Furthermore, when the NFκB activator LPS (Lipopolysaccharide) was used to treat SH-SY5Y cells for 16 hours, endogenous PINK1 protein levels were elevated compared to the untreated control by 26.77% ± 2.33%, 24.46 ± 2.59% and 83.57 ± 4.60% (*p* < 0.001) (Figure 4D), respectively.

Mitochondrial dysfunction plays a critical role in the development of PD and other neurodegenerative diseases such as Alzheimer’s disease [26]-[29] and multiple sclerosis [30],[31]. Mutations in several genes including UCHL1 [32], LRRK2 [33], PINK1 [12],[34], PARKIN [35] are associated with PD pathogenesis. PINK1 is a mitochondrial targeted protein and has been extensively studied because of its crucial role in autosomal recessive familial Parkinson’s disease [36] and protective role against mitochondrial dysfunction, oxidative stress and apoptosis [37],[38]. However, the regulation of PINK1 gene expression has not been well addressed yet [39]. For the first time, we studied the transcriptional regulation of the human PINK1 gene in the present study.

To investigate the transcription and translation of the human PINK1 gene, we first mapped the adenine 91 bp upstream of the first ATG in exon 1 as the transcription start site. Next we cloned the 1.8 kb fragment of 5’ UTR of the human PINK1 gene into a promoterless vector upstream of a reporter luciferase gene. The luciferase assay showed that this 1.8 kb fragment has strong promoter activity, indicating that the 1.8 kb fragment functions as the human PINK1 gene promoter. Subsequently, by deletion analysis we found that the 104 bp fragment from −78 to 26 serves as the minimal promoter region. The human PINK1 gene promoter does not have high GC content (<50%) like the human BACE1 and TMP21 promoter [40],[41]. Computer-based analysis revealed that the human PINK1 promoter contains several putative transcriptional factor binding elements such as MZF1, AP1F, CREB, NFκB and EGRF. Due to the importance of NFκB in PD, the potential regulation of PINK1 by NFκB was examined. There are four putative NFκB binding sites in the human PINK1 gene promoter and our results indicate that two of them physically bind with NFκB p65 with one strong binding site at the distal end. The effect of NFκB p65 on human PINK1 gene promoter was further confirmed by luciferase assay, where the overexpression of NFκB p65 elevated PINK1 promoter activity dramatically. Furthermore, we examined the effects of overexpression of NFκB p65 on PINK1 expression at the transcription and translation levels. Indeed, NFκB p65 increased the human PINK1 expression significantly at both the mRNA and protein levels. Due to the low expression level of the human PINK1 gene, few commercially available antibodies could detect the endogenous PINK1 clearly. We therefore cloned the human PINK1 plasmid, encoding a full-length PINK1 protein (~63 kDa) that is proteolytically processed into at least two shorter forms (~55 kDa and ~45 kDa), into a human dopaminergic neuroblastoma cell line (SH-SY5Y) [6],[7],[42]. In this cell line, we were able to detect three PINK1-related bands. Only the full-length PINK1 could be detected in the HEK293 cell line. This might be due to different expression levels and mitochondrial function between the different cell lines.

In this study, we clearly showed that NFκB significant upregulates PINK1 expression in both human and rodent cell lines, indicating that same regulation pathway exists in both human and rodents. It provides the strong evidence to support the use of rodent models to study the regulation of PINK1 expression *in vivo*, promoting the investigation of PD pathogenesis and drug development. Moreover, as SH-SY5Y cells are human dopaminergic cells and the dopaminergic neurons are mainly affected in PD patients, the results from SH-SY5Y cells can better represent the possible PINK1 regulation pathway in the brain of PD patients.

N-terminal proteolysis of FL-PINK1 produces two fragments, ∆1-PINK1 and ∆2-PINK1. After processing the proteolysis productions translocate to cytosol and undergo proteasome degradation [6],[7],[42]. Although the functional role of PINK1 in mitochondria or in Parkinson’s disease has not been well defined, PINK1 has been shown to confer a protective effect through preventing mitochondrial dysfunction, anti-apoptosis and promoting cell survival [43]. It has also been reported that FL-PINK1 accumulates in the brains of patients with sporadic PD or other synucleinopathies, and interestingly, the cleavage products of PINK1 are also increased in PD brains [44]-[46]. This accumulation of ∆-PINK1 might due to the upregulation of PINK1 expression in response to PD-related stress [44]. Given that truncated PINK1 can confer protection, increased cleavage of PINK1 may play a role in protecting neurons during PD pathogenesis. Our result showed that all of the three forms of PINK1 (FL-PINK1, ∆1-PINK1 and ∆2-PINK1) were elevated by overexpression or activation of NFκB signaling. Thus, the increase of PINK1 expression by NFκB activation could be neuroprotective. On the other hand, previous studies have suggested that the elevation of NFκB in dopaminergic neurons of PD patients is a reflection of the apoptotic and inflammatory state of the diseased brain [16]. These studies suggest that the activation of NFκB could be a double-edged sword in PD pathogenesis and the role of NFκB dysregulation in PD warrants further study.

## Conclusion

Our results demonstrate that PINK1 expression is tightly regulated at its transcription level and NFκB signaling is a positive regulator for PINK1 gene expression.

## Methods

### Primers and plasmid construction

To amplify the 5’-untranslated region of the PINK1 gene from DNA extracted from the human neuroblastoma cells SH-SY5Y, a forward primer (5’-cgcgagctcgttgcccaggctggtcttg) corresponding to −1799 bp of the transcriptional start site on exon 1 and a reverse primer (5’-cccaagcttcacaacaaacttggggcggtgcc) corresponding to +26 bp of the transcriptional start site were used. The PINK1 DNA fragment was cloned into a pGL3-basic vector upstream of a luciferase reporter gene (Promega) to construct pPINK1-A. Primers were designed to include restriction enzyme sites so that the PCR-amplified fragments can be inserted into the multiple cloning sites of the pGL3-basic vector. A series of deletion mutations (pPINK1-B, pPINK1-C, pPINK1-D, pPINK1-E, pPINK1-F, pPINK1-G, pPINK1-H and pPINK1-I) of PINK1 promoter were constructed utilizing primers listed as following: 26 F-cccaagcttcacaacaaacttggggcggtgcc, and 1799R- cccaagcttgttgcccaggctggtcttg; −795 F -ggcatggggatccaccatcttg, and -339 F-ccgctcgaggacctcgaatgctgccc; −103 F-ccgctcgagaaagtcactgctagaggc, and -50R-cccaagcttcgactcggcgcgtggggg; −78 F-ccgctcgagccagcatagcgcccccac, and -47 F -ccgctcgaggaactgccgcgggggccg, and -19 F-ccgctcgagccagcgcctgcgcctgcg, respectively.

### Switching mechanism at 5’end of RNA transcription (SMART) RACE cDNA amplification

Total RNA was extracted from SH-SY5Y cells using TRI reagent (Sigma) following the manufacturer’s protocol. SMART-RACE was performed using the SMARTer™ RACE cDNA Amplification Kit (Clontech) following the user’s manual. Simply, the first stand cDNA was synthesized from total RNA extracted from SH-SYHY cells with oligo (dT) primer in the presence of SMARTer IIA oligonucleotide (5’-aagcagtggtatcaacgcagagtacxxxxx (X is undisclosed base in the proprietary SMARTer oligo sequence). The SMARTer IIA oligonucleotide is able to anneal to the 5’-end of the first stand cDNA and serve as template to extend the 5’-end cDNA tail. A PINK1 reverse primer (5’-ccgaagcttgccctgcaagcgtctcgtgt) was specifically designed to recognize the +431 to +450 bp of human PINK1 gene downstream of the translation start site (ATG). The PCR products containing PINK1promoter region were amplified using SMARTer IIA oligonucleotide and PINK1 reverse primer and the first stand cDNA as template. The resulting PCR products were cloned into pcDNA4/myc-His A vector for sequencing and the first nucleotide linking with the adapter sequence was identified as the transcription start site of the human PINK1 gene.

### Cell culture, transfection, and luciferase assay

Human embryonic kidney HEK293, mouse neuroblastoma N2A and human neuroblastoma SH-SY5Y cell lines were cultured in Dulbecco’s modified Eagle’s medium (DMEM) containing 10% fetal bovine serum (FBS), 1 mM sodium pyruvate, 2 mM L-glutamine, 50 units/ml penicillin G sodium, and 50 μg/ml streptomycin sulfate (Invitrogen). All cells were maintained in a humidified 37°C incubator containing 5% CO_2_. 500 ng plasmid DNA per well of 24-well-plate for luciferase assay or 2 μg plasmid DNA per 35-mm-diameter plate for RNA extraction and Western blot analysis were used for cell transfection, respectively. For luciferase assay, pCMV-Rluc (Promega, USA) was co-transfected as a control to normalize the transfection efficiency. The cells were transfected using PEI reagent (Cat^#^. 23966, Polysciences Inc.) or Lipofectamine-™ 2000 reagent (Invitrogen). Cells were harvested 24 hrs after transfection and lysed with 100 μl 1× passive lysis buffer (Promega) per well. Firefly luciferase activities and Renilla luciferase activities of the same sample were sequentially assayed on a luminometer (Turner Designs Model 20/20) following the protocol of the Dual-luciferase Reporter Assay System (Promega). The firefly luciferase activity was normalized to the Renilla luciferase activity and the resulted value reflected the promoter activity.

### Electrophoretic mobility shift assay (EMSA)

HEK293 cells were transiently transfected with NFκB p65 and nuclear extracts were collected. Cells were rinsed and harvested with 1 × phosphate-buffered saline. After centrifugation, cell pellets were resuspended with 5 × volume of buffer A [10 mM HEPES pH 7.9, 10 mM KCl, 0.1 mM EDTA, 0.1 mM EGTA, 1 mM dithiothreitol (DTT), 0.5 mM phenylmethylsulfonyl fluoride (PMSF)]. Cells were pipetted up and down gently and maintained on ice for 15 minutes. The cell suspension was transferred to a Kontes all glass Dounce tissue grinder and ruptured by 10 strokes. 10% NP40 was added into the cell suspension for a final concentration of 0.5%. The samples were placed on ice for 15 minutes and stroked 5 more times. Crude nuclei were collected by centrifugation at 2000 g for 10 minutes and washed three times with buffer A containing 0.5% NP40 and resuspended in buffer C [20 mM HEPES pH 7.9, 0.4 mM NaCl, 1 mM EDTA, 1 mM EGTA, 1 mM dithiothreitol (DTT), 1 mM phenylmethylsulfonyl fluoride (PMSF), 10% Glycerol] at 4°C for 15 min. The samples were centrifuged at 12000 g for 4 min at 4°C, and the supernatant containing nuclear proteins was collected. EMSA was performed as previously described [40]. Four pairs of oligonucleotides containing the putative NFκB binding site on human PINK1 promoter region were synthesized for detecting the binding ability of NFκB to PINK1 promoter. The sequences of the oligos were listed as following: NFκB-54-35, forward, agtcggggaactgccgcggg and reverse, cccgcggcagttccccgact; NFκB-111-92, forward, gcaaagggaaagtcactgct and reverse, agcagtgactttccctttgc; NFκB-814-794, forward, ggcatggggatccaccatctt and reverse aagatggtggatccccatgcc; NFκB-1493-1474, forward, caaatgggaaattcatctat and reverse atagatgaatttcccatttg. The probes were labeled with IRDye-700 (IDT). Prior to incubation with nuclear extract, oligonucleotide probes were heated at 98°C for 5 minutes and annealed at 65°C for 10 minutes. 0.5 pmol of annealed probes were incubated with 2 μl of nuclear extract for 20 minutes at 22°C and the reaction mixtures were separated on a 4% Tris-glycine-EDTA gel in darkness. The mobility of probes on the gel was visualized using the LI-COR Odyssey (LI-COR Biosciences). For the competition assay, wild-type NFκB consensus oligonucleotides (forward: agttgaggggactttcccaggc, and reverse: gcctgggaaagtcccctcaact) and mutant NFκB consensus oligonucleotides (forward: agttgaggccactttcccaggc, and reverse: gcctgggaaagtggcctcaact) were used as positive and negative controls, respectively. For the super shift assay, the nuclear extract was incubated in 10 × EMSA binding buffer (100 mM Tris, 500 mM KCl, 10 mM dithiothreitol, pH 7.5) with monoclonal anti-NFκBp65 antibody (Sigma) for 10 minutes prior to the probe adding.

### Semi-quantitative RT-PCR

HEK293 cells and SH-SY5Y cells transfected with NFκB p65 were harvested 48 hours after the transfection and total RNA was extracted using TRI reagent (Sigma). Reverse transcription was sequentially performed using ThermoScript™ RT-PCR system kit following the manufacturer’s protocol (Invitrogen). The PINK1 gene specific primer PINK1 776 F-taccagtgcaccaggagaag and PINK1 984R- gcttgggacctctcttggat were used to amplify a 208 bp fragment of human PINK1 gene. The β-actin gene was also amplified using the forward primer (hActin-662fBamHI: cgaggatccggacttcgagcaagagatgg) and reverse primer (hActin-1124rXbaI: cagtctagagaagcatttgcggtggacg), which produced a 500 bp fragment. The PCR products were analyzed on 2% agarose gels.

### Western blot analysis

Western blot was performed as described previously [47],[48]. NFκB p65 plasmid DNAs (2 μg/35 mm dish) were transfected into HEK293 cells and SH-SY5Y cells. 48 hours after transfection, cells were harvested by RIPA-DOC buffer (1% Triton X-100, 0.1% sodium dodecylsulphate, 1% sodium deoxycholate, 0.15 mM NaCl, 0.05 M Tris–HCl pH 7.2, 0.5 mM phenylmethylsulfonyl fluoride) containing protease inhibitors (Roche). Cell lysates were separated on 10% Tris-glycine gel and proteins were transferred onto PVDF membrane, followed by primary antibody incubation overnight at 4°C. The primary antibodies were rabbit anti-PINK1 polyclonal antibody (1:500 dilution, Novus BC100-494), anti-p65 (1:1000 dilution, Cell Signaling), monoclonal anti-NFκB p65 antibody (1:1000 dilution, Sigma), and anti-β-actin antibody AC-15 (1:100, 000 dilution, Sigma). The protein bands were visualized using the LI-COR Odyssey (LI-COR Biosciences) after secondary antibody (goat anti-mouse, goat anti-rabbit, LI-COR Biosciences) labeling. The resulted protein bands were quantified by Image J software. The experiment was done in triplicate to minimize experimental errors.

## Competing interests

The authors declare that they have no competing interests.

## Authors’ contributions

XD and WS conceived and designed the experiments; XD, JT, QX, YW performed the experiments; XD, YW, TL and WS analyzed the data; FC, WS contributed reagents/materials/analysis tools; XD, YW and WS wrote the paper. All authors read and approved the final manuscript.
